# Cutting-Edge Perovskite-Based Flexible Pressure Sensors Made Possible by Piezoelectric Innovation

**DOI:** 10.3390/ma17174196

**Published:** 2024-08-24

**Authors:** Adeela Naz, Yuan Meng, Jingjing Luo, Imtiaz Ahmad Khan, Rimsha Abbas, Suzhu Yu, Jun Wei

**Affiliations:** 1 Shenzhen Key Laboratory of Flexible Printed Electronics Technology, Harbin Institute of Technology (Shenzhen), Shenzhen 518055, China; adeelanaz257@gmail.com (A.N.); 23s155096@stu.hit.edu.cn (Y.M.); 21b355005@stu.hit.edu.cn (J.L.); imtiazkhattak533@gmail.com (I.A.K.); rimsha.abbas31@gmail.com (R.A.); 2 School of Materials Science and Engineering, Harbin Institute of Technology (Shenzhen), Shenzhen 518055, China; 3 State Key Laboratory of Advanced Welding and Joining, Harbin Institute of Technology (Shenzhen), Shenzhen 518055, China

**Keywords:** perovskite, sensing mechanism, flexible pressure sensor, piezoelectric properties

## Abstract

In the area of flexible electronics, pressure sensors are a widely utilized variety of flexible electronics that are both indispensable and prevalent. The importance of pressure sensors in various fields is currently increasing, leading to the exploration of materials with unique structural and piezoelectric properties. Perovskite-based materials are ideal for use as flexible pressure sensors (FPSs) due to their flexibility, chemical composition, strain tolerance, high piezoelectric and piezoresistive properties, and potential integration with other technologies. This article presents a comprehensive study of perovskite-based materials used in FPSs and discusses their components, performance, and applications in detecting human movement, electronic skin, and wireless monitoring. This work also discusses challenges like material instability, durability, and toxicity, the limited widespread application due to environmental factors and toxicity concerns, and complex fabrication and future directions for perovskite-based FPSs, providing valuable insights for researchers in structural health monitoring, physical health monitoring, and industrial applications.

## 1. Introduction

The development of modern technologies has been rapid. Because of this, sensor technology is becoming more and more common in society and has garnered the attention of researchers, particularly in the field of pressure sensors [1]. Pressure sensors in flexible electronics are a crucial intersection of material sciences, system engineering, signal processing, and device technology incorporating artificial intelligence [2]. Pressure sensors are an essential member of flexible electronics and have seen significant advancements due to their flexibility, compatibility with large areas, and cost-effective processing methods [3]. These sensors can be evaluated based on a number of criteria, including sensitivity, hysteresis and working range [4], recovery time and response [5,6] and stability. Due to the fact that every application has its own specific requirements for sensor characteristics, it is vital to have an understanding of the fundamental parameters that are utilized in the selection of pressure sensors. Piezoelectric [6,7,8], piezoresistive [9,10,11], capacitive [12,13,14], and triboelectric [15] sensing mechanisms are some important types of pressure sensors.

FPSs are being improved through strategies like developing pressure-responsive materials, enhancing device structures, and improving manufacturing techniques. Micro-structuring of the sensing layer has gained significant attention over the past 20 years [16,17,18]. Some of the substrates that are commonly used in FPSs are polyethylene terephthalate (PET), polyimide (PI), polydimethylsiloxane (PDMS) and polyethylene naphthalate (PEN). Thermoplastic polymer (PDMS) is a popular choice for FPSs due to its stability, mechanical flexibility, and compatibility.

Research on piezoelectric materials for FPSs has been primarily focused on various types of flexible materials [19], such as metals, polymers, and carbonaceous nanomaterials, including silver nanowires (AgNWs), polyvinylidene fluoride (PVDF), polyvinylidene fluoride-trifluoroethylene (PVDF-TrFE), potassium sodium niobate (KNN, (K,Na)NbO_3_), carbon nanotubes (CNT), carbonitrides (Mxene), and graphene. Nanomaterials are gaining scientific interest due to their potential in wearable sensing applications [20,21,22]. They offer flexibility, biocompatibility, ultrahigh conductivity, commercial availability, low cost, and numerous processing methods for coating fibers and fabric, depending on the material consistency. Driven by the increasing demand for artificial skins, flexible display screens [23], human machine interaction [24,25], medical health [26,27], the Internet of Things (IoT) [28], electronic skins (e-skins) [29,30,31], electronic textiles (e-textiles) [32], flexible touch displays [22], soft robotics [33], and energy-harvesting devices [34] have emerged as applications in the field of FPSs in the last few years. The traditional pressure sensors in micro-electro-mechanical systems (MEMS) are typically made of rigid materials such as metal, semiconductors, and piezoelectric crystals. Techniques such as inkjet printing [35], magnetron sputtering [36], electrospinning [37], screen-printing [38], and dip-coating [39] are using these materials to fabricate FPSs and can potentially achieve outstanding efficiency in terms of mass fabrication and low measurement errors. FPSs offer lightweight, conformable designs for easy integration into various surfaces and wearable devices, enhancing user comfort and applications in soft robotics, health monitoring, and smart textiles. However, they face challenges like limited durability, lower sensitivity, and environmental factors.

There have been a great number of recent review articles that have described FPSs. Most of these articles are skewed toward particular applications in fields like robotics, AI (artificial intelligence) [2,40], biomedicine [41,42], and healthcare [43,44]. These articles explore sensor structure, function, and specific materials, with researchers focusing on developing sensing materials like nanocomposite, carbon-based polymers, and Mxene [10,43,45].

**Figure 1:**
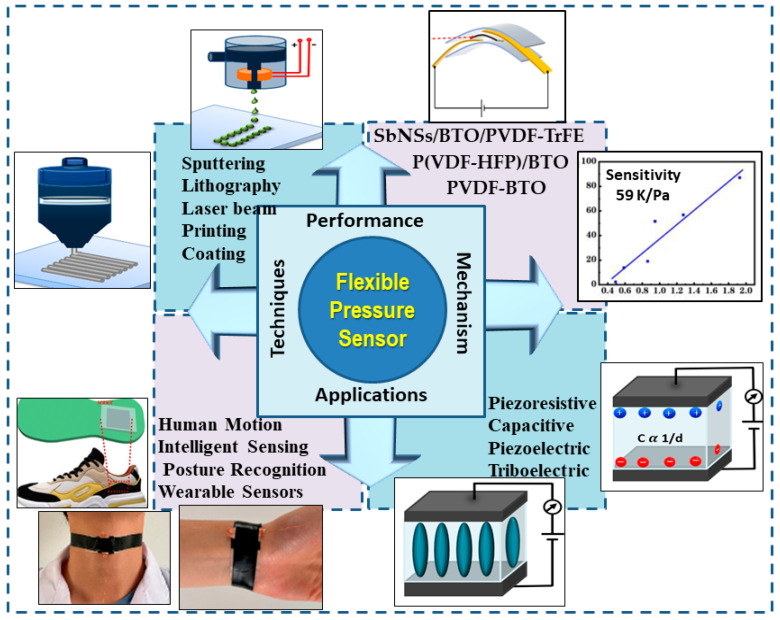
A comprehensive view of the current research on flexible pressure sensors.

In order to transfer the technology of FPSs from the laboratory to the real world, these articles have provided engineers with a variety of perception functions. For example, research dedicated to microstructure modeling, fabrication, and sensor fabrication techniques [46,47] has been essential for the manufacturing of such devices. On the other hand, there is no review article that has provided a summary of the piezoelectric performance associated with perovskite materials for FPSs. A comprehensive view of this review is explained in Figure 1.

This review provides a comprehensive analysis of perovskite materials used in FPSs, focusing on their piezoelectric properties and fabrication techniques. It highlights the unique focus on piezoelectric performance in perovskite materials, offering practical insights for researchers and engineers. The review also discusses the anticipated difficulties and opportunities associated with perovskite-based FPSs, aligning with future performance in applications like structural health monitoring. The review addresses FPS technology from multiple angles, including material science, device engineering, and system integration. Recent advancements include self-powered sensors, capacitive and piezoresistive mechanisms, and triboelectric mechanisms. The review also highlights the need for lead-free piezoelectric ceramics due to environmental concerns.

Perovskite-based flexible pressure sensors have evolved significantly over time, particularly in their unique electrical and mechanical properties. Advances in material science and fabrication techniques have made them reliable and efficient, with applications in healthcare, robotics, and wearable technology. Future research aims to enhance their performance and to reduce production costs.

## 2. Important Components for Flexible Pressure Sensors

Important components for FPSs include the piezoelectric materials, electrodes, casing, insulating materials, signal conditioning circuitry, connectors, and wires.

### 2.1. Sensing Elements

Piezoelectric materials like crystals, ceramics, and polymers generate electrical charges in response to pressure, serving as sensing elements in pressure sensors. They exhibit rapid and dynamic responses, making them suitable for monitoring in real time and for high-frequency measurements.

### 2.2. Electrodes

The working principle of an electrode in a pressure sensor depends on the type and sensing mechanism. Capacitive pressure sensors use changes in capacitance to measure pressure variations, with electrodes placed on either side of a flexible diaphragm. The sensor measures the capacitance change and converts it into a pressure reading using calibration and signal processing techniques. Piezoresistive pressure sensors use changes in electrical resistance to measure pressure variations, often using a Wheatstone bridge configuration. The electrodes’ design, materials, and configuration depend on the sensor’s application, pressure range, sensitivity requirements, and environmental conditions.

### 2.3. Casing

The casing of a pressure sensor is a crucial component that provides protection, isolation, pressure transmission, mounting capabilities, sealing, environmental compatibility, and an appropriate size and form factor. It shields the internal components of the sensor from external hazards, prevents interference from external factors, and transmits applied pressure to the sensing element. The casing also serves as a sturdy structure for secure attachment to various components, ensuring a watertight seal. The casing’s design also considers the overall size, shape, and form factor of the sensor, ensuring its reliability, accuracy, and longevity in diverse operating conditions.

### 2.4. Insulating and Encapsulation Material

The insulating material in pressure sensors is crucial for maintaining integrity, functionality, and accuracy. It provides electrical isolation, prevents leakage, protects against environmental factors, ensures dielectric strength, and ensures compatibility with sealing techniques. Common materials include polymers, ceramics, and specialty coatings.

### 2.5. Signal Conditioning Circuitry

The signal conditioning circuitry in a pressure sensor process amplifies electrical signals, including via signal amplification, gain adjustment, filtering, linearization, temperature compensation, calibration, analog-to-digital conversion, and output interfaces. It optimizes sensor sensitivity, reduces measurement errors, corrects temperature-induced drift, and facilitates seamless integration with control systems.

### 2.6. Connectors and Wiring

The connector and wiring in a pressure sensor are essential for transferring electrical signals between the sensor and external devices. They ensure secure connections, proper signal transmission, and compatibility with sensor specifications, electrical standards, and industry guidelines.

### 2.7. Calibration Elements

Calibration elements in pressure sensors ensure accuracy, reliability, and consistency. They include reference standards, calibration procedures, adjustments, calibration curves, zero-point calibration, span calibration, periodic calibration, and traceability. These elements account for drifts, aging effects, environmental changes, and manufacturing variations.

## 3. Sensing Mechanisms for Flexible Pressure Sensors

**Figure 2:**
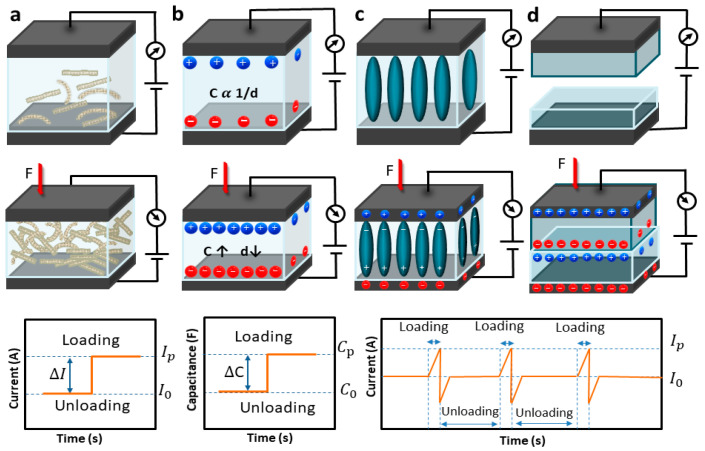
Schematic illustration of four common transduction mechanisms: (**a**) piezoresistive, (**b**) capacitive, (**c**) piezoelectric and (**d**) triboelectric.

FPSs can be classified as having piezoelectric [6], piezoresistive [43,48], capacitive [11,49,50], or triboelectric mechanisms [51]. Each sensing mechanism has unique properties due to the sensor structure and active materials, which will be dissected in this section (Figure 2).

### 3.1. Piezoelectric Mechanism 

The applied stress is converted into electric voltage using piezoelectric sensors that offering high sensitivity and high voltage output [52] through macroscopic polarization, allowing them to detect variations in external pressure through variations in voltage. By exerting mechanical pressure from the outside against a piezoelectric material, these sensors produce a piezoelectric effect, resulting in a charge separated into two distinct spatially positive and negative states. The separation of negative and positive charges occurs when the material is distorted by external pressure. The effect of external forces is determined by analyzing potential differences and determining their impact. Piezoelectric sensors have a high sensitivity and quick response time, making them suitable for detecting dynamic pressures like slips and vibrations. They are passive and suitable for various applications, but they are not suitable for detecting static pressures due to their decreasing voltage output over time.

Piezoelectric materials with high piezoelectric coefficients, low weight, dimensional stability, workability, chemical stability, and chemical inertness, including ceramic lead zirconate titanate (PbZrTiO_3,_ PZT) and polymer PVDF, are suited for dynamic tactile sensing [53,54,55]. Currently, it is still a difficult task to build self-powered pressure sensors that have the ability to detect both static and dynamic pressures. Polymers and inorganic materials, such as PZT [56,57], zinc oxide (ZnO) [58], PVDF [59,60,61], and barium titanate (BTO, BaTiO_3_) [62], are commonly used in piezoelectric applications. Shahzad et al. [63] developed PVDF-TrFE and modified graphene oxide fiber membranes GO-P(VDF-TrFE) for piezoelectric pressure sensors. GO’s large surface area enhances charge generation and storage, improving its electrical properties. These sensors can withstand 80 °C and generate signals under 10 Pa, making them suitable for high-vacuum products. Shirley et al. [64] created a piezoelectric textile pressure sensor using a layer structure of ZnO sandwiched between conductive woven fabrics. The sensor effectively monitors forces of uniaxial pressure, twisting, and bending, and it can be attached to cloth for clinical purposes and to track human movements.

### 3.2. Capacitive Mechanism

The capacitance variation of dielectric materials between conductive electrodes is used by a capacitive sensor [65]. It typically has a sandwich structure with an elastic dielectric layer and two parallel electrodes. The sensor’s capacitance is related to the dielectric layer’s permittivity, electrode area, and distance. External pressure causes deformation, enabling mechanical pressure detection through capacitance transduction. Takamatsu et al. created large significant-area of fabric for capacitive pressure sensors using conductive polymer die-coating and automatic looming machines. The sensors measure capacitance changes between stripe electrodes under pressure, with an output capacitance change of 1.37 F. Using the edge effect, we can compute the capacitance of the capacitor. The sensor’s initial capacitance is small because of the distance between electrodes. When the external pressure is applied, the dielectric layer compresses, bringing the internal components closer into contact. Furthermore, Gurarslan et al. [66] created a conductive wool-knitted fabric coated with AgNW for a touch-based sensor capable of monitoring body motions like joint movement and respiration. To improve sensitivity, large electrode and dielectric deformations under low stress can be achieved using elastomeric electrodes or microarray architecture.

### 3.3. Piezoresistive Mechanism

A piezoresistive pressure sensor has a layered-by-layered structure with two electrodes and a layer of piezoresistive material. Piezoresistive sensors have been extensively researched due to their simple device structure, low operating voltage, low energy consumption, wide working range, ease of fabrication, and signal collection. Variations in resistivity can occur in semiconductors or composites due to pressure-induced changes or changes in the interparticle distance. In response to applied external pressure, the intermediate piezoresistive material deforms, changing the device resistance. This allows for the determination of external pressure changes [67]. Qi et al. [68] developed a highly sensitive pressure sensor using a 1D wearable core made of nanofiber yarn coated with carbon nanotube-embedded polyurethane nanofibers. The sensor has a large surface area and elasticity, resulting in high sensitivity (16.51 N^−1^), a wide sensing range (0.003–4.9 N), and a short response time (0.03 s). This sensor can be used for human motion capture and physiological signal monitoring.

### 3.4. Triboelectric Mechanism

The triboelectric effect occurs when materials make contact with one another, generating charges on their surfaces. Triboelectric pressure sensors have undergone extensive investigation. When pressure is applied, the combined effect of contact on both electrostatic and electrical induction results in the generation of voltage and polarization. This phenomenon is common in daily life, such as when dressing, combing hair, and walking. Researchers have discovered that surface charges can be produced from mechanical motion via this electrostatic induction, converting energy into electrical energy. Triboelectric materials, including positive and negative ones, are also needed. Metals like Al, Ni, Ag, and Cu are commonly used as positive electrodes. Negative materials like PDMS and PVDF are often coated with fibers or textiles.

Guo et al. [69] developed an all-fiber hybrid piezoelectric-enhanced triboelectric material using silk fibroin and PVDF nanofibers on conductive fabrics. This innovative design offers outstanding electrical performance, flexibility, and air permeability and can be customized for clothing. We will also compare these mechanisms in Table 1.

## 4. Piezoelectric Property of Ceramic Perovskite Material for Flexible Pressure Sensors

Pressure sensors use solid piezoelectric materials like BTO, KNN [74], PZT [75], PbTiO_3_ (PT) [76], etc. However, piezoelectric ceramics or polymer composites can combine the high-voltage electromechanical coupling and electrical properties of piezoelectric ceramics with polymers’ chemical stability, flexibility, and stretchability. PI, PDMS, PVDF and polyacrylonitrile (PAN) are extensively utilized as matrices [61,77].

Since the discovery of quartz crystal in 1880, piezoelectric materials have garnered a significant amount of attention. Piezoelectric ceramics are considered to be classical functional materials in electronic devices. Since its discovery in 1954, PZT-based materials have been extensively employed in piezoelectric ceramics for commercial applications.The previous progress in the piezoelectric properties of perovskite materials (Table 2).

PZT is restricted worldwide, despite its piezoelectric properties. Thus, a non-Pb PZT competitor is urgently needed. Specifically, (PbZr_1−x_Ti_x_O_3_, PZT)-based ceramics are popular for their high T_c_ 80–320 °C and their incredible piezoelectric coefficient (d_33_ ≈ 200–750 Pcn^−1^). PZT- and Pb-based perovskite composites are preferred for their outstanding dielectric, electromechanical, and piezoelectric properties and their stability, making them widely used in actuators and sensors. The global piezoelectric device market reached USD 25.1 billion in 2019 and is expected to grow 6.2% annually. However, lead-free piezoelectric research is crucial due to global legislation banning lead-based materials in electronic products, increasing the market and promoting the utilization of lead-free ceramics.

In the past, newly developed piezoelectric ceramics have been driven by advancements in sensors, ultrasonic transducers, and actuators. Since the beginning of the twenty-first century, interest in the research, advancement, and development of lead-free piezoelectric ceramics has grown [25]. The global market significance for these ceramics reached ≈ USD 172 million in 2019. This value is predicted to increase to approximately USD 443 million in 2024, with an annual growth rate of ≈ 20.8% [78]. The characteristics of lead-free materials to improve the flexibility and performance of FPS will be examined.

**Table 2:** Piezoelectric properties of different perovskite materials.

Material	d_33_ (pCN^−1^)	T_c_ (°C)	K_p_	References
(Bi_0.5_Na_0.5_) TiO_3_	72.9	325	0.168	[79]
0.01% Co-BNT	105	25	-	[80]
0.03% Eu-BNT	110	324	-	[81]
0.5%ZnO-BNT	110	-	0.17	[82]
0.985BaTiO_3_–0.015Sr(Cu_1/3_Nb_2/3_)O_3_	333	97	-	[83]
NN-BT 10%	147	235	-	[84]
BNT-BT_x_, x = 0.04	87	230	0.21	[85]
BNT-BT_x_, x = 0.02	78	265	0.20	[85]
BNT-BT_x_, x = 0.08	112	250	0.13	[85]
BNT-BT_0.06_	103	262	-	[86]
Sm-BNT-BT_0.06_	202	280	0.30	[87]
Eu-BNT-BT_0.06_	149	310	-	[88]
Er-BNT-BT_0.06_	9	303	0.13	[89]
0.5 Ta-BNT-BT_0.06_	170	260	-	[90]
La-BNT-BT_0.06_	167	280	0.30	[91]
BNT-BT_0.06_-xBFO, x = 0.03	80	290	-	[92]
(BaCa)(Hf-Ti)O_3_	336	106	-	[93]
BCZT	225	77.2	-	[94]
Ba_0.93_Ca_0.07_Ti_0.975_Sn_0.025_O_3_	216	122	-	[95]
BCSnT	478	63	-	[96]
Ba_0.88_Ca_0.12_Ti_0.94_Sn_0.06_O_3_	220	68	-	[97]
Ba_0.95_Ca_0.05_Ti_0.95_Sn_0.05_O_3_	431	72	-	[98]
(Nb_x_Sb_0.08_)O_3_-xLiTaO_3_-xBaZrO_3_	365	170	0.45	[99]
(K_x_Na_x_)_0.98_Li_x_(Nb_0.77_Ta_0.18_Sb_0.05_)O_3_	413	225	0.50	[100]
K_0.5_Na_0.5_NbO_3_	160	423	-	[101]
((KNaLi)_1+x_ NbSbTa)O_3_ (x = 0.01)	310	-	0.6	[102]
0.95K_0.46_Na_x_Nb_0.95_Sb_0.05_O_3_-xBi_0.5_(Na_0.82_K_0.18_)_0.5_ZrO_3_	465	233	0.43	[103]
K_0.5_Na_0.5_NbO_3_-Bi_0.5_Na_0.5_TiO_3_)-LiSbO_3_	250	339	0.29	[104]
(K_x_Na_x_)_0.95_Li_0.05_Nb_1−x_Sb_x_O_3_-BaZrO_3_	425	197	0.50	[105]
((K_0.48_Na_x_)(Nb_0.95_Sb_x_)O_3_)-xBNKLZr	380	290	0.46	[106]

### 4.1. Bismuth Ferrite (BiFeO_3_)

Bismuth ferrite (BFO, BF) stands out as an ideal material for pressure sensors due to its unique combination of piezoelectric, ferroelectric, and multiferroic properties. Earlier studies have indicated its excellent performance in BF-based materials [107,108,109,110].

Bismuth ferrite–barium titanate, also known as BFBT, is a new material that has emerged that does not have the presence of lead. It possesses a high T_c_ > 400 °C and ferroelectric properties of ~40 μC/cm^2^. Among the numerous lead-free material have shown promising electrical and mechanical properties, BF-based ceramics are replacing lead-based materials, that give higher *T*_c_ of BF enables it to solubilize with BaTiO_3_ (BT), SrTiO_3_ (ST), and other compounds. when it added with an ABO_3_ structure, creates an MPB, which is an established procedure for obtaining high piezoelectric properties. For example, Lee et al. [48] were able to obtain a piezoelectric coefficient (d_33_) of 402 pCN^−1^ and a Curie temperature (T_c_) of 454 °C in the morphotropic phase boundary (MPB) region by quenching gallium (Ga)-doped BF-0.33BT. Furthermore, Habib et al. [111] were able to achieve a d_33_ of 436 pCN^−1^ and a piezoelectric strain (d_33_*) of 550 pm/V by quenching samarium (Sm)-doped BF-0.33BT ceramics. Zhang et al. [112] studied manganese (Mn)-doped Bi_0.675_La_0.025_FeO_3_–0.3BTO. The synergistic composition model and new poling method resulted in excellent piezoelectricity (d_33_ = 436 pCN^−1^ and d_33_* = 550 pmV^−1^) and a high T_c_ of 450 °C. The piezoelectricity improvement technique can be used to optimize the functional properties of other lead-free materials.

BFO’s flexibility and compatibility with various substrates facilitate its integration into flexible electronics, maintaining its performance even under bending or stretching conditions. Moreover, its stability and robustness at different temperatures and environmental conditions improve the reliability and durability of the sensors.

### 4.2. Sodium Titanate (SrTiO_3_)

Sodium titanate (STO, ST) stands out as an exceptional material for pressure sensors due to its remarkable piezoelectric and dielectric properties, which are crucial for high-sensitivity and accurate pressure detection. Its perovskite structure contributes to excellent mechanical flexibility, allowing the sensor to conform to various configurations without affecting its performance. A comprehensive investigation has been conducted on the microstructure and electrical properties of lead-free (1−x)BF–xST (0.32 ≤ X ≤ 0.44) ceramics near the MPB. The final results show that these ceramics had a high polarization value of 51.2 μCcm^−2^ with a 72 pCN^−1^ piezoelectric coefficient [49]. Bismuth sodium titanium oxide(BNT)-ST had a significant impact on the electromechanical properties of the material, showing a 127 pCN^−1^ piezoelectric coefficient [113]. The phase shift behavior and electrical properties of KNN-100ST were also investigated, which showed that the highest piezoelectric coefficient value of 130 pCN^−1^ was achieved at x = 0.05 [114]. Additionally (Bi_0.5_Na_0.5_)TiO_3_-BTO-STO thin films were deposited onto Nb-STO, which provided the largest strain level and the highest piezoelectric coefficient of 209 pmV^−1^. For the BNBT-0.1ST sample, a significant dynamic piezoelectric coefficient was observed, which was calculated to be d_33_^*^ = 350 pCV^−1^ [115]. Furthermore, a very large normalized strain of d_33_* = 488 pmV^−1^ was obtained at x = 0.28 on BNST100x. A d_33_ of 667 pmV^−1^ corresponds to a high normalized strain [116]. STO has stability under varying environmental conditions, ensuring reliable operation in diverse applications from wearable technology to industrial monitoring. Additionally, its compatibility with flexible substrates and straightforward integration into microelectronic systems make it a versatile choice for FPSs, promising enhanced durability and responsiveness. Through the utilization of the sol-gel electrospinning technique, nanofiber membranes that were doped with stannum (IV) of SrTiO_3_ (SSTO) were successfully manufactured. A long-term bending stability of ≈100 cycles was demonstrated, along with quick response and recovery characteristics of 12–30 ms, which is higher than that of other piezoresistive sensors that are based on polymer fibers (for a detailed comparison of the various perovskite composites, see Table 2).

### 4.3. Barium Titanate (BaTiO_3_)

The invention of BTO during World War II has been widely regarded as a remarkable advancement in piezoelectric materials. In comparison with other conventional ferroelectric materials, BTO has garnered the most attention due to the fact that it possesses exceptional dielectric, ferroelectric, optical, piezoelectric, and other significant properties [117]. Using a solid-state reaction, the lead-free Ba_0.95_Ca_0.05_Ti_0.95_Sn_0.05_O_3_ (BTCS) ceramic achieved a large value of the piezoelectric coefficient, d_33_, which was ≈ 431 pCN^−1^, as well as a d_33_^*^ that was ≈ 403 pmV^−1^ [98]. Pure BTO and 0.85Ba(Ti_0.89_Sn_0.11_)O_3_–0.15(Ba_0.7_Ca_0.3_)TiO_3_ (BTS–0.15BCT) ceramics were able to achieve a d_33_ ≈ 740 pCN^−1^ and a dielectric constant of ≈ 6800 [118]. Ferroelectric systems with a pseudobinary system of (BaCaTiO_3_-Ba (Sn, Ti)O_3_), (Ba(Zr, Ti)O_3_-(Ba, Ca)TiO_3_) and ((Ba, Ca)TiO_3_-Ba(Hf, Ti)O_3_) was reported to exhibit d_33_ values that fall within the 450–700 pCN^−1^ range [119,120]. The enhancement in the electromechanical response, on the other hand, comes at the expense of a significant reduction in the Curie point. Take, for instance, the composition that exhibits a d_33_ between 600 and 700 pCN^−1^ and has a T_c_ of around 40 °C [121]. Li et al. [122] achieved d_33_ = 165 pCN^−1^ for the 0.7BF–0.3BT ceramics. The electrical properties of the ceramics were determined to be *d*_33_ = 210 pC/N and *P*_r_ = 31.2 μC/cm^2^. However, it is very difficult to form complex shapes with inorganic ceramic materials, which are inherently mechanically brittle and easily broken during operation, which restricts their application in wearable devices. This is because of the fact that they are easily broken during operation. The creation of piezoelectric nanocomposites through the combination of polymers and nanoparticles has been demonstrated to be an effective method for resolving this issue. The excellent mechanical and high flexibility properties of PVDF and its copolymers have resulted in a significant amount of interest in these materials.

Additionally, BTO exhibits strong mechanical flexibility, allowing it to maintain its performance under various bending and stretching conditions, which is crucial for the durability and reliability of flexible sensors. Furthermore, its chemical stability and compatibility with a variety of flexible substrates enhance its integration into diverse sensor designs.

### 4.4. Potassium Sodium Niobate (K_0.5_Na_0.5_NbO_3_)

The past few decades have seen an increase in the popularity of KNN ceramics due to the fact that they are environmentally friendly and possess properties that can be adjusted. Among the potential substitutes for PZT are piezoelectric ceramics based on textured KNN [123,124]. The piezoelectric parameters of sodium niobate ceramics and pure phase potassium, which are k_p_ = 0.40 and d_33_ = 125 pCN^−1^, are significantly lower than those of PZT ceramics that have T_c_ = 415 °C. The growing interest in KNN ceramics began a few years ago. Lin et al. [125] synthesized a textured (1−x)-(K_0.5_Na_0.5_)(Nb_0.96_Sb_0.04_)O_3_-x(Bi_0.5_Na_0.5_)HfO_3_ KNNS-BNH ceramic and successfully achieved both high piezoelectric properties (d_33_ = 474 pCN^−1^) and excellent temperature stability. Additionally, KNNBC-xBNH ceramics manufactured by Fan et al. [126] using solid-phase methods exhibited a favorable T_c_ of 333 °C and piezoelectric coefficient of d_33_ = 272 pCN^−1^. The lead-free KNNS-0.035BNH ceramics exhibited remarkable piezoelectric properties such as a d_33_ of 436 pCN^−1^, a k_p_ value of 0.56, a d_33_* value of 604 pmV^−1^, and a T_c_ value of 253 °C.

Its high piezoelectric coefficients ensure sensitive and accurate pressure detection, which are crucial for applications ranging from medical diagnostics to wearable technology. Additionally, KNN’s inherent mechanical flexibility allows it to be integrated into flexible substrates without compromising its piezoelectric performance, making it ideal for next-generation development of FPSs that require both high adaptability and also sensitivity to various forms and surfaces. 

**Figure 3:**
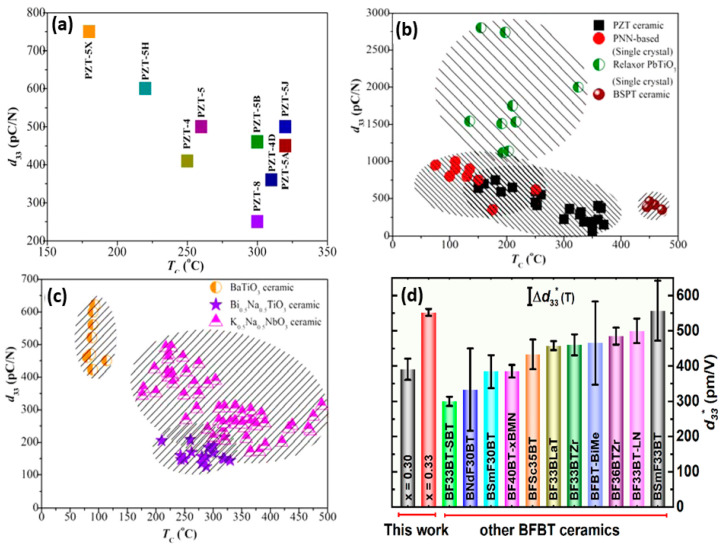
Presentation of the (**a**) d_33_ and Curie temperature of PZT-based, (**b**) lead-based, (**c**) and lead-free ceramics, sourced from several publications. (Reprinted with the permission from [127]. copyright 2015, American Chemical Society.) (**d**) Graph showing the d_33_* for BFO—xBTO (x = 0.30 and 0.33), analysis of temperature stability, and the d_33_* of lead-free BFBT ceramics. (Reprinted with permission from [111]. copyright 2023, Materials Science & Technology).

The comparison of the d_33_ coefficient of reported ceramic-based materials is also discussed below (Figure 3).

## 5. Performance of Perovskite Materials in Flexible Pressure Sensors

Perovskite materials are popular in photovoltaics, optoelectronics, and sensing due to their high carrier mobility, adjustable bandgap, and robust piezoelectric response. Combining them with flexible substrates can create accurate and responsive pressure sensors, enhancing their applications.

**Figure 4:**
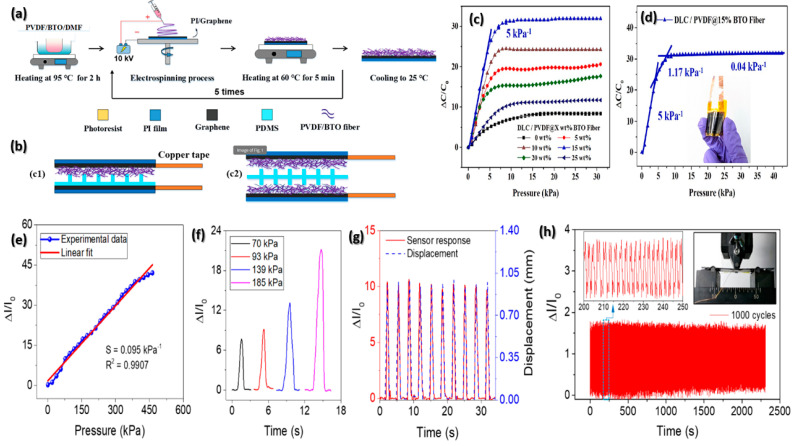
(**a**) Diagram showing the steps involved in creating PVDF-BTO electrospun fibers using electrospinning. (**b**) Diagrammatic representation of the flexible pressure sensor assembly with single- and double-sided microstructures. (**c**) Comparing the sensitivity of sensors using fibers with varying BTO ratios and double-layer microcylinders (DLC) as a dielectric layer and (**d**) with DLC-BTO fibers at different pressures of 0–5, 5–7.5, and 7.5–40 kPa. (Reprinted with permission from [128]. copyright 2022, Measurement). Fully integrating nanometer-thick perovskite with a mechanoluminescence (ML) device results in a robust, flexible, and self-powered mechanoluminescent perovskite pressure sensor. (**e**) Current reaction to pressures applied to the sensor. (**f**) Time-varying sensor response. (**g**) The bending for ten cycles. (**h**) Sensor response as a result of cyclic bending tests; an enhancement of the sensor signal at a 200–250 s range is displayed in the inset. (Reprinted with the permission from [129]. copyright 2020, ACS Applied Nano Materials).

The composites of PVDF-BTO are ideal for FPSs due to their fast response, high sensitivity and release times, low detection limit, durability, enhanced dielectric properties, and ease of fabrication. These sensors have a sensitivity of 5 kPa^−1^, allowing for greater capacitance variations under pressure. They also have a low limit of detection of 0.11 Pa, making them ideal for delicate pressure-sensing applications. The composites are easy to fabricate, involving electrospinning and casting methods. The PVDF-BTO composite-based sensors show high durability, withstanding over 10,000 compression cycles and 5000 bending cycles without signal degradation. This robustness ensures long-term reliability for flexible and wearable applications. They have been successfully applied in various practical tests, including breath monitoring, real-time body movement tracking, pulse monitoring, and acoustic vibration detection. These properties collectively contribute to the superior performance of PVDF-BTO composites in FPSs, making them highly suitable for advanced applications in wearable technology and electronic skin systems (Figure 4a–d) [128].

Furthermore, perovskite materials are ideal for FPSs because of their high sensitivity, self-powered operation, durability, flexibility, efficient light harvesting, large sensing range, and ease of fabrication. Perovskite-based sensors demonstrate notable sensitivity. For instance, a mechanoluminescent perovskite sensor exhibits a sensitivity of 0.095 kPa^−1^, allowing it to detect minimal pressure changes effectively. These sensors can detect minimal pressure changes, operate without external power, and demonstrate stable performance, with consistent signals observed over 1000 cycles, indicating the potential for long-term reliability. Their flexible nanometer-thick layers make them suitable for applications requiring bending and deformation without the loss of functionality with wide sensing range, up to 460 kPa (Figure 4e–h) [129].

**Figure 5:**
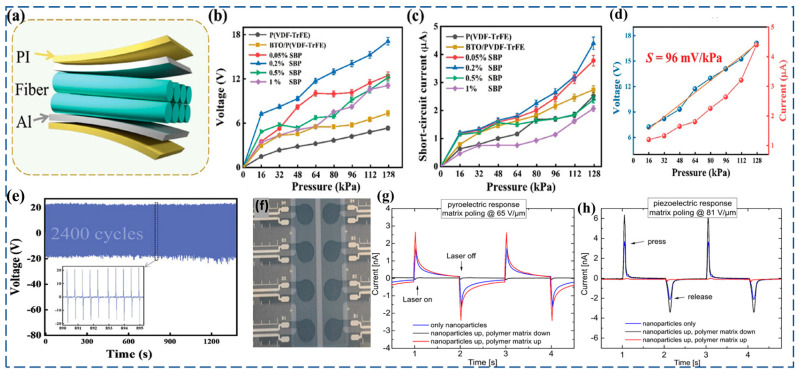
(**a**) Illustration device structure of FPPSs; (**b**,**c**) SbNSs/BTO/PVDF-TrFE (SBP) composite-fiber short-circuit currents, voltages, and dynamic force compressive pressures at 2 Hz; (**d**) Output currents vs. voltages according to the applied pressure; (**e**) Output stability of FPPS-SbNSs doped in 2Hz and BTO doped in 128 kPa. (Reprinted with permission from [130]. copyright 2023, Advanced Electronic Materials.) (**f**) Sensor view with PET film by using screen-printed technquie. (**g**,**h**) Current reactions to piezoelectric and pyroelectric stimulation following AC poling with a field amplitude that compensates for the pyroelectric or piezoelectric responses. (Reprinted with permission from [131]. copyright 2020, ACS Applied Materials and Interfaces).

Wang et al. [130] were able to successfully prepare highly sensitive flexible piezoelectric pressure sensors (FPPSs) by utilizing the enhancement effect of nanoparticles of BTO and antimony(Sb) nanosheets(SbNSs) on the piezoelectric properties of nanocomposite fibers P(VDF-TrFE) by using electrospinning. Electrostatic interactions between polymers and inorganic fillers produce effect to increasing the amount of polar *β*-phase that is present in the nanocomposite fibers through the presence of P(VDF-TrFE). The mixing of BTO and SbNSs resulted in a significant enhancement of the piezoelectric output of FPPSs. BTO-P(VDF-TrFE) nanocomposites are ideal for flexible pressure sensor applications due to their enhanced piezoelectric properties, mechanical flexibility, high sensitivity, thermal stability, and ability to decouple pressure and temperature sensitivities (Figure 5a–d).Additionally, P(VDF-TrFE)-NaBiTi_2_O_6_(BNT) nanocomposites are ideal for FPSs due to their piezoelectric and pyroelectric properties. These properties enable the material to respond to pressure and temperature changes, making it versatile. The material can be formulated into screen-printable pastes, making it suitable for large-scale production. BNT is a lead-free alternative to traditional ferroelectric materials, making it more sustainable. The selective poling of the P(VDF-TrFE) matrix and BNT nanoparticles allows for precise control over the material’s sensing properties, reducing cross-sensitivity. The combination of these properties makes them suitable for different applications, including wearable devices and artificial skin (Figure 5e–h) [131].

**Figure 6:**
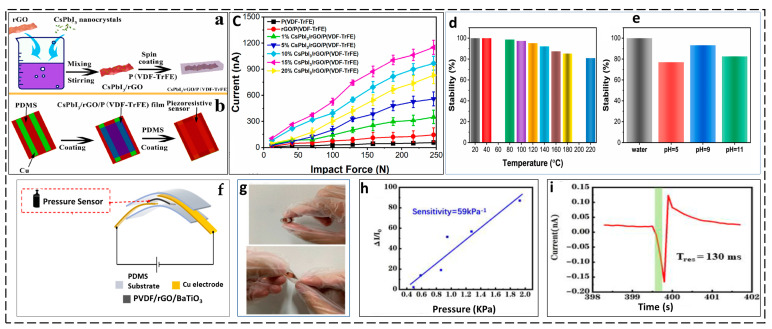
(**a**) CsPbI_3_/rGO/P(VDF-TrFE) film precursor solution preparation. (**b**) Fabrication of a flexible sensor. (**c**) CsPbI_3_/rGO/P(VDF-TrFE) flexible pressure sensor output currents at varying impact forces. (**d**) Pressure sensor stability at different temperatures after 1 h. (**e**) Pressure sensor stability in water, sodium hydroxide, and sulfuric acid after 48 h. (Reprinted with the permission from [132] copyright 2022. Physica B: Condensed Matter). (**f**) Diagram of a PVDF/rGO/BTO-based pressure sensor for flexible electronic skin (FES), displaying its multi-layered structure and characteristics. (**g**) The FES under curling conditions. (**h**) The sensitivity of the FES. (**i**) The response time. (Reprinted with permission from [133]. copyright 2021, Organic Electronics).

The CsPbI_3_/rGO/P(VDF-TrFE) composite offers several advantages for flexible pressure sensor applications. It enhances piezoelectric and photoelectric properties, resulting in a uniform and spatially spread mesh composite film. This leads to an open-circuit voltage of 12.5 V and a circuit current density of 425 nA, which are substantial improvements in piezoelectric pressure sensors. The composite also shows superior stability in water, thermal, and acid–base conditions, making it ideal for long-term performance and reliability. Additionally, it allows for self-powered operation, reducing the need for external power sources and enabling more autonomous and sustainable sensor systems. These characteristics make the CsPbI_3_/rGO/P(VDF-TrFE) composite an ideal material for developing high-performance FPSs (Figure 6a–e) [132].

Furthermore, the PVDF/rGO/BTO composite is a versatile material for FPSs. It combines rGO, PVDF, and BTO to enhance sensitivity, thermal stability, and electrical conductivity. The composite’s thin film structure ensures a high sensitivity of 59 kPa^−1^ and a quick response time (130 ms), with a high output current of about 1200 Pa under specific pressures. The composite is synthesized by the near-field electrohydrodynamic direct-writing (NFEDW) technique, creating a micro/nanofiber structure that enhances the pressure sensitivity and response speed. The composite’s well-dispersed BTO particles create a two-phase structure with a smooth interface, further improving sensitivity (Figure 6f–i) [133].

**Figure 7:**
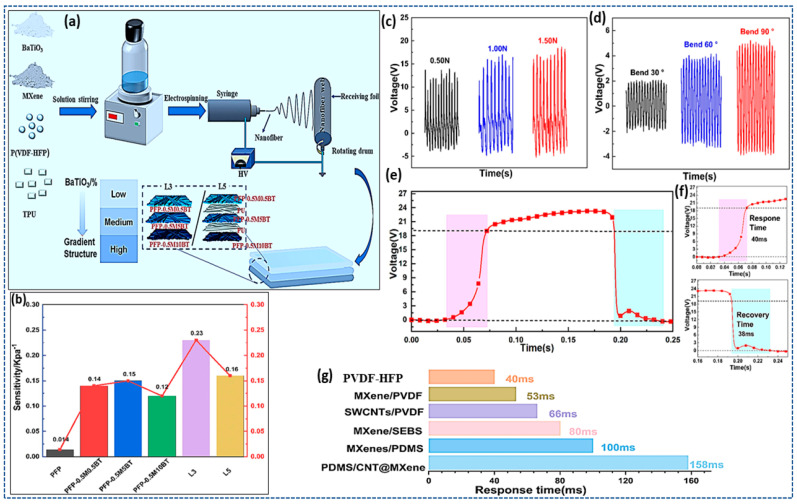
(**a**) Poly(vinylidene-hexafluoropropylene) P(VDF-HFP) composite-fiber preparation technology. (**b**) Sensitivity of multilayered composite fibers. (**c**) Three-layer gradient composite film output voltage under different forces. (**d**) Different angles of bending. (**e**,**f**) Three-layer gradient composite film output voltage recovery time and response time. (**g**) Examination the response time of different materials from other studies. (Reprinted with permission from [134]. copyright 2023, Diamond and Related Materials).

He et al. [134] describes that the P(VDF-HFP)/MXene/BTO composite materials show promise in flexible pressure sensor applications because of their enhanced sensitivity, high piezoelectric output, strong mechanical properties, improved dielectric performance, fast response time, and efficient production process. The multi-layer composite-fiber membrane-based sensor shows a sensitivity that increase to16.4 kPa^−1^ as compared with pure P(VDF-HFP) at a low-pressure range of <1 kPa, which increases by 1.5 times when compared with the PFP-0.5M5BT nanofiber, achieving 0.23 kPa^−1^. Its high output voltage is ideal for applications requiring significant signal generation from minimal force. The composite’s dielectric properties are enhanced by MXene, allowing for better energy storage and signal transduction. The composite’s simple and efficient preparation process using the electrospinning method makes it viable for large-scale production applications (Figure 7).

Furthermore, Liu et al. [135] focused on the use of MXene in piezoelectric materials to improve the performance of sensing devices. They prepared composite films of BT/MXene/PVDF-TrFE using electrospinning. The composite film, containing 0.15 weight percent MXene, achieved a ꞵ-phase crystal content of 81.04% and a stable piezoelectric response even after 5000 compression cycles. This research aimed to enhance the performance of such sensors.

## 6. Fabrication Techniques for Flexible Pressure Sensors

**Figure 8:**
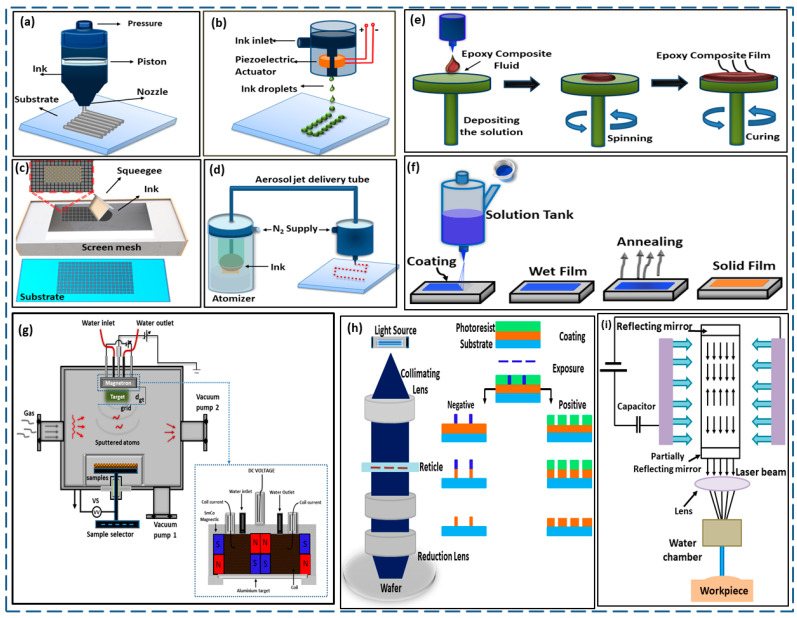
Schematic presentation of printing techniques. (**a**) Direct ink writing. (**b**) Ink jet printing. (**c**) Screen printing. (**d**) Aerosol jet printing. (**e**) Spray coating process. (**f**) Spin coating process. (**g**) Working principle of the magnetron-sputtering deposition process. (**h**) Process of photolithography. (**i**) Laser beam machining setup.

**Figure 9:**
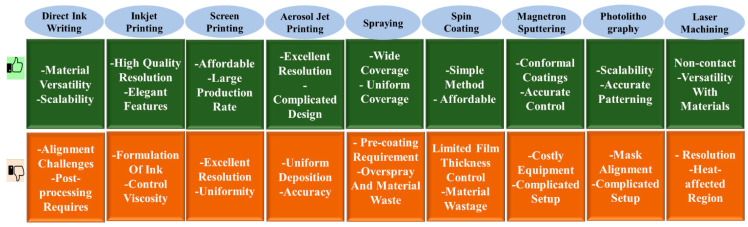
Assessment of techniques to fabricate FPSs [147,148].

It is possible to fabricate an FPS sensitive layer using different type of techniques, depending on the structure and design of the sensors, the materials used, and the application that is intended for the sensor. Some of the most common methods of fabrication that are utilized for FPSs are as follows (Figure 8); their advantages and limitations are also compared in Figure 9.

Advanced printing methods have shown potential in flexible sensor fabrication, enabling high resolution and complex sensor patterns, unlike other methods. Three-dimensional (3D) printing methods simplify complex objects and pattern them from computer-aided designs, aiding in the manufacturing of electronic gadgets like sensors, robots, and wearables. These techniques enable the creation of novel structures with high resolution. Direct ink writing (DIW) is a manufacturing technique used to create FPSs. It involves extruding conductive ink onto a substrate, creating complex three-dimensional structures. DIW offers simplicity, scalability, and versatility, but its resolution is limited by the nozzle diameter, and maintaining ink stability can be challenging [136,137]. Inkjet printing (IJP) is a high-resolution technique used for fabricating FPSs by depositing conductive ink droplets onto a substrate. It offers high resolution, digital control, and material efficiency, but challenges include ink formulation compatibility and uniform droplet size for sensor performance [138]. Screen printing is another printing technique that is cost-effective, and it is a robust technique for fabricating FPSs’ sensitive layer. It can handle various materials and is ideal for large-area production. However, it has limitations in resolution and layer uniformity, impacting the pressure sensor performance [139]. Aerosol jet printing is also a precise and flexible technique for fabricating flexible pressure sensor layers. It uses conductive ink mists to create intricate patterns on various substrates, including metal nanoparticles, polymers, and graphene. Aerosol jet printing is feasible for printing onto various substrates, including those with complex geometries and flexible materials. The advantages of this technique include its high resolution, ability to create intricate patterns, and minimal material waste. However, it requires precise control and may incur high equipment costs [140].

Spray coating is a scalable technique for depositing the FPS sensitive layer. It involves spraying conductive materials onto substrates, forming a uniform film. Common materials include silver nanowires, carbon nanotubes, and conductive polymers. It is easy to implement, covers large areas, and is cost-effective. This is not needed for the ion-selective membrane/PVC protective layer since the solvent, THF, evaporates faster, allowing continuous substrate coating. Patterns are made using shadow masks that match each layer’s pattern. Similar to optical lithography shadow masks, these prevent the material from being sprayed in unwanted locations. Changing the shadow mask is enough to swiftly deploy a new pattern. Small-scale spray coating with a stationary setup requires a lot of material. If the spray head is not changed throughout the sample, only the center has a uniform layer thickness. This can be avoided by using non-stationary spraying equipment or by scaling up the fabrication process, such as in roll-to-roll operations [141]. 

Spin coating is another technique used to create thin, uniform films for FPSs. It involves depositing conductive material onto a substrate, then spinning it at high speeds. This method offers precise control over the film thickness but is limited to planar substrates [142]. Magnetron sputtering is a PVD technique used to create high-purity, uniform thin films for FPSs. It involves bombarding a target material with ions in a vacuum chamber, depositing materials like metals, oxides, and nitrides. Despite its advantages, it can be slow and costly. This capability is useful for large-scale manufacturing with high throughput. Additionally, it provides good adhesion between the flexible substrate and the thin layer. Sputtering creates strong connections between the deposited material and the substrate. This helps the sensor retain mechanical integrity under strain or distortion. However, magnetron sputtering for sensor manufacturing has some drawbacks. High-energy plasma may harm the flexible substrate or change its mechanical properties. To avoid these issues, substrate materials and process conditions need to be carefully chosen. The operation and maintenance of sputtering equipment are challenging and expensive, requiring specific skills [143]. Photolithography is also a high-resolution patterning technique used to fabricate intricate designs in FPSs. It transfers geometric patterns onto a photoresistant layer, etching them into the substrate. Despite its complexity and cost, it remains a critical technique in microfabrication [144]. Laser machining is a precision and controllable technique used to fabricate FPSs using metals, polymers, and composites [145]. It creates complex designs with minimal waste and is compatible with various substrates. However, it is limited by the laser power and material properties, and its use can lead to high equipment costs [146].

## 7. Application of Perovskite-Based Flexible Pressure Sensors

Perovskite-based materials can be synthesized through low-temperature synthesis processes that are both cost-effective and environmentally friendly. Among the electronic and optoelectronic applications that could benefit from the large-scale production of perovskites are diodes, photodiodes, high-efficiency light-emitting diodes, solar cells, and sensors [149,150]. Perovskites are promising candidates for the production of these materials.

### 7.1. Human Motion Detection

**Figure 10:**
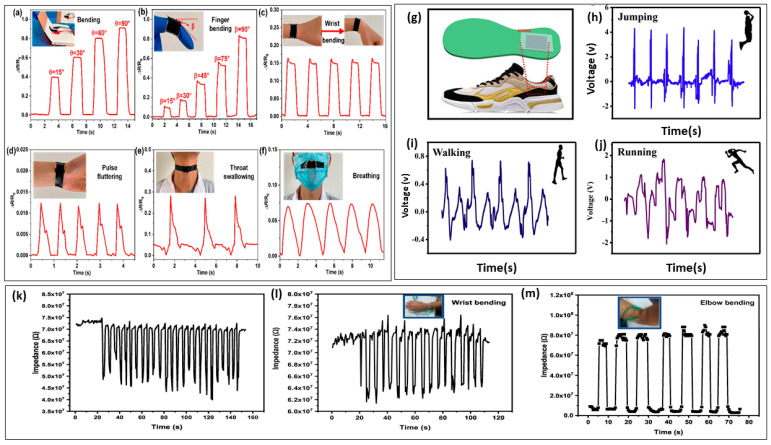
Flexible SSTO-nanofiber-based sensors are used to measure the following: (**a**) notebook bending; (**b**) finger bending, (**c**) wrist bending, (**d**) the radial pulse, (**e**) throat swallowing, (**f**) nose breathing. (Reprinted with permission from [151]. copyright 2021, ACS Applied Materials & Interfaces). (**g**) The PDA@BTO/PVDF piezoelectric flexible pressure sensor integrated into shoes, and the output voltages produced by (**h**) jumping, (**i**) walking, and (**j**) running. (Reprinted with permission from [152]. copyright 2020, Sensors and Actuators A: Physical). The application of a PU-BTO-rGO flexible pressure sensor for calculating motion by (**k**) touching with a finger, (**l**) wrist bending, and (**m**) elbow bending. (Reprinted with permission from [153]. copyright 2023, Diamond and Related Materials).

Pressure sensors are pivotal in the field of human motion detection due to their sensitivity and adaptability. Gao et al. [151] fabricated stannum-IV-doped SrTiO_3_ (SSTO) by using sol-gel electrospinning. SrTiO_3_-nanofiber piezoresistive sensors have great potential for monitoring human motion because of their high sensitivity and low pressure range, their rapid response and recovery, their flexibility, and their good stability of structure. The sensor chip is utilized to monitor the bending angles; the ratio of R/R_o_ increases with the increase in the angle of bending, which demonstrates the high flexibility and also sensitivity of pressure sensors. The chips contain the sensor could be used to measure fluctuations of the human pulse with a periodicity of 75 beats per minute. This is the typical frequency for a typical human being, and it is possible to observe even minute variations. A sensor that was attached to the throat and nose was also able to detect repeated swallowing and breathing motions in an accurate and instantaneous manner without any delay in time. The aforementioned findings provide evidence that the piezoresistive sensor based on SSTO nanofibers possesses low-pressure high sensitivity, rapid response and quick recovery, good flexibility, and tolerance to a variety of environments. As a result, these sensors have the potential to be utilized in smart wearable devices, medical and healthcare applications, and in the chemical industry (Figure 10a–f) [151].

One major problem is the fabrication of FPSs with a broad detection range and good sensitivity. Traditional piezoelectric materials, like BTO, while excellent in terms of their piezoelectric properties, suffer from inherent brittleness and mechanical instability, making them less suitable for flexible applications. Moreover, ensuring consistent performance under dynamic and strenuous conditions such as those encountered in human motion monitoring is difficult. To overcome these challenges, Yan et al. [152] used a facial solution-casting technique to combine BTO with PDA and PVDF, which has been employed for wearable applications. By utilizing this PDA-modification strategy, it is possible to enhance the diffusion of the BTO/PVDF matrix and also to decrease the number of interface hole defects that exist between components. As a consequence of this, the 17% weight percent PDA@BTO/PVDF sensor demonstrated a remarkable output voltage of 9.3 V and a rapid response time of 61 ms. This was a significant improvement in comparison with the PVDF/BTO/PVDF composite counterparts. Furthermore, the sensor, in its capacity as an energy supplier, has the capability to generate a maximum power of 0.123 μWcm^−2^ despite in the presence of a high load resistance 70 MΩ. When the flexible pressure sensor was incorporated in the shoe sole, voltages were produced for jumping, walking, and running (Figure 10g). The findings indicated that the output voltage due to walking was ≈ 1 V, which was significantly lower than the voltages for jumping (≈ 6.5 V) and running (≈ 3.8 V) (Figure 10h–j). The reason for this is that the impact force that the human foot exerts in the course of walking is relatively low and gentle, whereas the impact force that is exerted during jumping and running is significantly higher. According to the findings presented above, the sensor possesses a significant amount of potential for use in applications involving human motion detection and wearable devices.

Graphene oxide was synthesized by Madbouly et al. [153] using a modified Hummer method, and the BTO-rGO nanocomposite was obtained through the co-precipitation method. Due to its high sensitivity, rapid response, fast recovery, reliability, and stability, the sensor that was examined has the potential to be useful in human activity monitoring.

A PU-BTO-rGO flexible pressure sensor was used to measure touching, wrist bending, and hand bending. The sensor showed high sensitivity, response, and recovery times when touched and removed. It also detected wrist bending and elbow bending, with the impedance variation approximated. The sensor’s response time was short, and the impedance change was identical before the bend. It was also capable of detecting pressure at various pressure levels with a high sensitivity of 2.63 kPa^−1^ and rapid response time of 560 ms (Figure 10k–m). These sensors have potential applications in monitoring routine human activities [153]. In comparison with other sensors that are based on polyurethane (PU), the proposed sensor possesses superior compressibility, a high response, and a broad range of pressure detection, and it is characterized by a quick response and recovery time.

**Figure 11:**
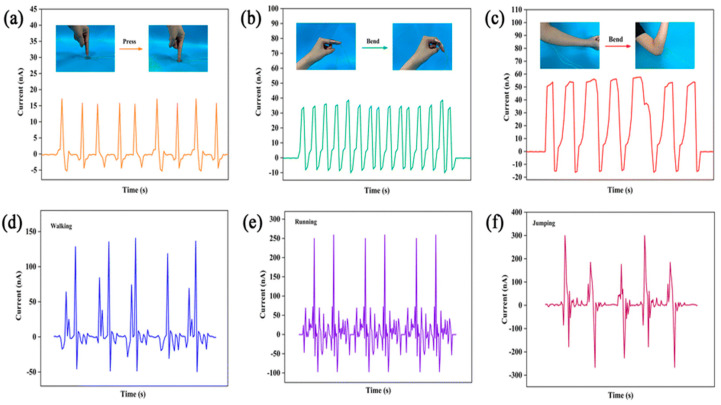
The use of the BTO/MXene/PVDF-TrFE flexible pressure sensor to detect human motion monitoring of the (**a**–**f**) index-finger release voltage, finger flex–release, elbow flex–release, walking, running, and jumping, respectively. (Reprinted with permission from [135] Copyright 2023, Materials Chemistry C).

BTO/MXene/PVDF-TrFE film is a wearable technology for monitoring human body motion. The sensor is attached to different parts of the human body, demonstrating high competence in identifying point pressure–release and flexion–release. The sensor also demonstrated remarkable capacity for bending and releasing when applied to joints. The sensor was affixed to the entire body to detect moments during everyday activities, with a detection current of approximately 130 nA when walking, 250 nA when running, and 300 nA when jumping. The composite pressure sensor exhibited a superior reaction speed and sensing range from 0.2–400 kPa [135] (Figure 11).

It could be used to identify difficult human motion, specifically in weight-bearing exercises for athletes. This innovative hybrid material maintains high piezoelectric performance, flexibility, and durability even under repeated mechanical stress. The sensor, which can monitor various human movements, has a broad detection range and quick response time. Its stability is maintained over 5000 compression cycles and shows no degradation in output voltage even after simulated sweat conditions. BTO is an optimal material for FPSs due to its exceptional properties, mechanical robustness, and environmental friendliness [135].

Wufan et al. [154] fabricated a superhydrophobic flexible pressure sensor that was developed using porous MAPbBr3/PVDF-TFSI sensing film with an EPDM coating. The sensor demonstrated excellent performance, including high sensitivity, a low detection limit, high precision, and a rapid response/recovery time. Its superhydrophobicity and thermal stability make it suitable for various applications, including wearable systems. The sensor accurately recognized human physiological signals and performed well in wind speed monitoring. This sensor could be a promising tool in wearable systems or in wind monitoring.

### 7.2. Intelligent Sensing and Recognition Applications

It is difficult to design self-powered sensors that are stretchable and skin-conformal in order to achieve intelligent sensing and posture recognition. AI is revolutionizing the development of smart pressure sensors, improving their functionality, performance, and applications. It can process real-time data, predict maintenance needs, calibrate sensors, optimize performance, and enhance sensitivity. AI can facilitate IoT integration, enable adaptive control systems, create customized solutions, detect faults, and optimize power consumption.

An all-in-one stretchable elastomer–piezoelectric pressure sensor (ASPS) with excellent sensitivity and durability and that can operate across a broad pressure range (10–900 kPa) with good linearity and rapid response times has been produced by a multi-force blending and melting method, in addition to the synergistic piezoelectricity of BTO. Its silicone components provide excellent skin conformality, making it comfortable and unobtrusive for applications like human–machine interaction and continuous health monitoring.

**Figure 12:**
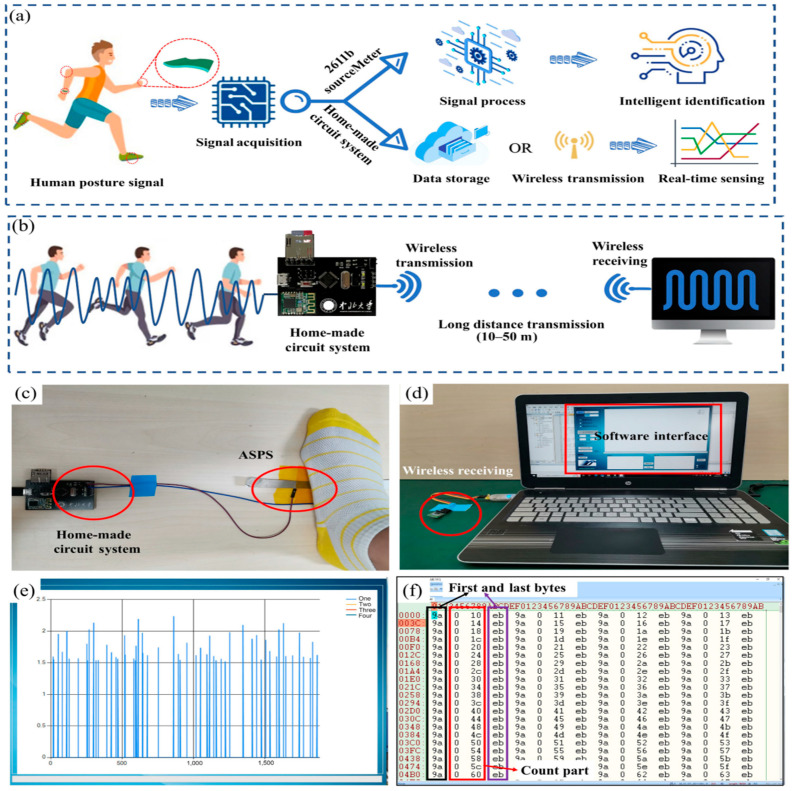
Applications of intelligent sensing: (**a**) System flow recognition. (**b**) Homemade circuit-system operating schematic. (**c**,**d**) Software interface, the wireless transmitted module, and circuit module. (**e**) Collection of the signal in the software interface. (**f**) Assessment of storage reliability. (Reprinted with from permission [155]. copyright 2023, Nano Research).

The ASPS demonstrates high sensitivity, with a voltage output of 0.93 V/10^4^ Pa and a current output of 4.92 × 10^−4^ Pa at a pressure range of 10–200 kPa (Figure 12). Furthermore, it has outstanding longevity, withstanding over 10,000 cycles without seeing any appreciable performance reduction [155]. The ASPS can be integrated with a homemade circuit for signal acquisition and wireless transmission and with an artificial intelligence algorithm for signal processing, enhancing its potential for wearable technology applications.

### 7.3. Wearable Applications

To be more specific, in order for wearable sensors to be successful, they need to have a low cost and incorporate outstanding elasticity, long-term stability, and accuracy, and the individual’s physiological and biological state must be constantly tracked in real time [156]. STO is a versatile material that can be used in pressure sensors. Additionally, when the sensor is positioned on the knee, it will generate various voltages depending on the activity; for example, walking (2 V), jumping (7.6 V), and kicking (2.6 V). This is also the case when the sensor is placed on the insole of the shoe. Nevertheless, the shoe insole produces a lower voltage of six volts in comparison to the knee and back, which produce eight volts. This is due to the fact that, initially, the sensor demonstrates a better response for folding and twisting than it does for tapping. In this case, the shoe insole functions as a touching mechanism, while the other two mechanisms act as bending or folding. By placing the EC10S + PNy11 pressure sensor in the pocket, it is possible for the pocket to function as a smart pocket, thereby prevent pickpocketing. When a person places their hand inside a pocket that has the sensor attached to it, the sensor will produce a voltage that is greater than one volt. Theft can be detected in the future by integrating software that is specifically designed for the purpose of catching it. Furthermore, the EC10S + PNy 11TES has the potential to be utilized in healthcare applications, including in smart chairs and beds, among other applications. The sensor displays a low voltage at 0.4 V when the individual is lying in the center of the bed. On the other hand, when the individual is moving their body like coma patient, like when they move toward the edge of the bed, the sensor displays a very high voltage of 0.6 V.

**Figure 13:**
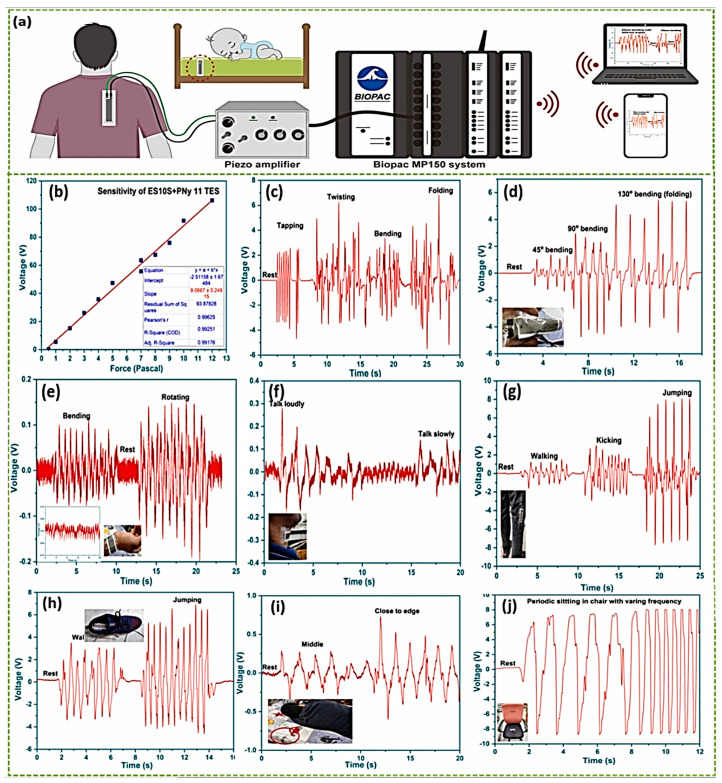
(**a**) The experimental configuration for the EC10S + PNy 11 TES human activity measurement. (**b**) The sensor sensitivity. (**c**) Sensing performance such as twisting, tapping, bending, and folding, and (**d**,**e**) When the sensor serves as a wrist band or finger ring. (**f**) The intensity of sound when the sensor is attached to the neckband. (**g**,**h**) Detecting and distinguishing between motions such as kicking, walking, and jumping. (**i**) Identifying the sleeping posture when the sensor is fixed to the edge of bed. (**j**) Applications for smart chairs in healthcare. (Reprinted with permission from [157]. copyright 2023, Nano Energy).

STO can be incorporated into Ecoflex, creating composite films that maintain sensor flexibility while enhancing its performance. The addition of STO enhances the dielectric properties of the composite film, resulting in increased triboelectric output performance. STO-based composites improve the performance under load, achieving a maximum voltage output of 270 V and a short-circuit current of 7.2 µA at 12.5 N. These sensors are ideal for real-time applications in healthcare, motion detection, and smart wearables. Their high sensitivity of 9 VPa^−1^ makes them effective for precise pressure measurements (Figure 13) [157].

The above work on the performance of flexible pressure sensors is summarized in Table 3.

## 8. Challenges and Future Perspectives

Perovskite-based FPSs have potential but face several challenges. These include material instability, mechanical flexibility and durability, integration with flexible substrates, piezoelectric stability at high temperatures, sensitivity vs. dynamic range, toxicity and environmental impacts, manufacturing scalability, encapsulation, and post-processing. These issues limit their widespread application due to environmental factors, toxicity concerns, and the complexity of fabricating sensors. To overcome these issues, researchers are developing novel materials and methods to enhance their stability, mechanical durability, and integration onto flexible substrates while ensuring environmental safety and cost-effective manufacturing processes. This requires continued research and innovation to reach the full potential of perovskite-based FPSs.

Innovations in material science are expected to improve the mechanical durability of perovskite sensors, enabling them to withstand repeated bending and stretching without performance degradation.

In order to maximize the sensing capabilities of perovskite materials for a variety of applications, it is essential to exercise precise control over the morphology and structure of these materials. Both the morphology and structure of these materials have an excellent impact on the optical and electrical properties, and they also show amazing results in stabilizing the properties that they possess. A number of factors, including crystallographic orientation, grain size, and surface area, have the potential to influence the sensitivity, selectivity, and response time of these types of materials. For example, large surface areas improve sensitivity by providing more active positions for identifying analytes. On the other hand, smaller grain sizes can enhance the response time by enabling electron transport and faster charge transfer. Both of these factors contribute to the overall performance of the material. It is possible that perovskites could become a promising prospect for the application of flexible pressure sensors if deep knowledge of the interaction between preparation processes and characteristics and precise control over morphologies is obtained. In order to maintain their sensing capabilities, FPSs must be able to withstand repeated bending and stretching. Perovskite materials, despite their flexibility, have the potential to crack or delaminate when subjected to continuous stress, which can have an impact on sensor performance.

In spite of the fact that perovskites have a great deal of potential in sensing applications, the fact that they are unstable and toxic gives rise to significant difficulties. Materials that are based on perovskite are susceptible to degradation when exposed to oxygen and moisture, which can reduce the amount of time that they can be used effectively. In order to find a solution to this problem, researchers are actively investigating new approaches that can improve the stability of materials based on perovskite sources. Furthermore, the presence of lead in certain materials based on perovskite is a significant cause for concern because of the potential harm that it could cause to both people and the environment from its presence. As a result, the development of materials based on perovskite that do not contain lead is particularly important for expanding their use in a variety of applications [11]. By giving higher priority to the development of materials that are both safer and more stable, it will be possible to enhance the full potential of perovskite-based materials.

The first step in advancing the development of sensors is the synthesis of materials that can detect pressure, which is also an area that is undergoing continuous improvement. The development of novel materials and structures that have multiple outstanding parameters, make use of a large area, and have a straightforward manufacturing process is one of the challenges. For example, multifunction sensing presents a challenge. While integrating flexible pressure sensors with other various kinds of sensors is a popular method, it does come with some drawbacks, including a complicated structure and the possibility of manufacturing processes that are incompatible with each other [158]. There is also a promising opportunity in the development of new materials that can respond to and decoupling multiple stimuli within a single sensing unit. Another challenge is to ensure that the electrodes and sensing components have a strong electrical and physical connection. This connection is necessary in order to prevent the sensor from delaminating when it is bent/stretched. Post-processing techniques, like encapsulation, are also essential for highly sensitive sensors. These techniques have the ability to maintain the original compressive state of sensing materials, thereby reducing the amount of disturbance that occurs.

When it comes to the design, fabrication, and development of flexible pressure sensors, this review will be of significant reference value. According to feedback on the limitations of the FPSs that are currently in use, we believe that continuous attention should be paid to the following aspects in order to improve the quality of the sensor as a whole.

Perovskite-based flexible pressure sensors have evolved significantly over time, with a focus on their unique electrical and mechanical properties. Advances in material science and fabrication techniques have made them reliable and efficient, with applications in healthcare, robotics, and wearable technology. Future research aims to enhance their performance and to reduce production costs.

## 9. Conclusions

Flexible pressure sensors (FPSs) have the potential to be used in pressure-induced strain gauges in order to efficiently monitor the structural health of buildings, automobiles, bridges, etc. The explicit study in this paper was needed to explore the efficiency and performance of FPSs made using different materials and fabrication techniques. The review explores FPS technology from various perspectives, highlighting advancements like self-powered sensors, capacitive and piezoresistive mechanisms, and the need for lead-free piezoelectric ceramics due to environmental concerns.

FPSs that are based on perovskite have shown promise over the course of the years, particularly in the fields of wearable and flexible portable electronic devices. As a result of their high sensitivity, flexibility, piezoelectricity/piezoresistivity, large strain properties, and tunable electrostriction and electronic properties, these materials have become the most desirable materials for use in a wide range of fields, which include health monitoring, human–computer interactions, and robotics. However, they face challenges like limited durability, lower sensitivity, and environmental factors.

In conclusion, the distinctive structure of perovskite materials enables them to exhibit a high degree of sensitivity to mechanical pressure. This, in turn, enables these sensors to detect a wide range of pressures with accuracy and precision. Their excellent properties and performance of perovskite-based FPS will assist researchers to create new perovskite compounds with enhanced or tailored properties for specific applications.

Furthermore, these sensors are adaptable, can conform to various surfaces, and are able withstand bending, making them suitable for wearable devices, flexible electronics, structural health monitoring, aerospace applications, marine ecosystem monitoring, and military infrastructure.

## Figures and Tables

**Table 1:** Comparison of mechanisms of flexible pressure sensors.

Type	Mechanism	Advantages	Limitations	Example	References
Piezoresistive	Resistance change	Simple design, high sensitivity	Hysteresis, temperature dependence	Textile-based FPS	[70]
Capacitive	Capacitance change	High sensitivity, low power	Complex circuitry, external interference	Flexible capacitive pressure sensors	[71]
Piezoelectric	Electric charge generation	High sensitivity, fast response	Limited to dynamic pressure, complex fabrication	Ultrahigh sensitivity FPS	[72]
Triboelectric	contact electrification	High output voltage, simple structure	Wear and tear, environmental sensitivity	FPS based on bionic-microstructures	[73]

**Table 3:** A summary of the reported performance of flexible pressure sensors.

Materials	Fabrication Techniques	Sensitivity	Response Time	Pressure Range	Operating Voltage	Cycles	Ref.
PVDF-BTO	Electrospinning	5 kPa^−1^	-	0.11 Pa	-	5000	[128]
SbNSs/BTO/PVDF-TrFE (SBP)	Electrospinning	96 mV kPa^−1^	2 ms.	128 kPa	17.1 V	2400	[130]
P(VDF-TrFE)-NaBiTi_2_O_6_(BNT)	Screen printing	good	-	-	12.5 V	-	[131]
CsPbI_3_/rGO/P(VDF-TrFE)	Spin coating	excellent	-	-	self-powered operation	-	[132]
PVDF/rGO/BTO	Direct ink writing	59 kPa^−1^	130 ms	-	-	-	[133]
P(VDF-HFP)/MXene/BTO	Electrospinning	0.23 kPa^−1^	-	<1 kPa	-	500	[134]
Stannum-IV-doped SrTiO_3_	Sol-gel electrospinning	high	-	rapid response	-	-	[151]
PDA@BTO/PVDF	Facial solution-casting method	-	61 ms	-	9.3 V	-	[152]
PU-BTO-rGO	Hummer method	2.63 kPa^−1^	560 ms	0–60 kPa	-	500	[153]
BTO/MXene/PVDF-TrFE	Electrospinning	0.2–400 kPa	56 ms	0.2–400 kPa	-	5000	[135]
ASPS	Mixing	excellent	-	10–900 kPa	0.93 V/104 Pa	10,000	[155]

## Data Availability

The original contributions presented in the study are included in the article, further inquiries can be directed to the corresponding authors.
